# Obstetrics Viewed from a Gynecological Standpoint1Read before the Obstetrical Section, Buffalo Academy of Medicine, September 25, 1900.

**Published:** 1900-11

**Authors:** Charles E. Congdon

**Affiliations:** Buffalo, 1034 Jefferson Street


### OBSTETRICS VIEWED FROM A GYNECOLOGICAL STANDPOINT.[1]

By CHARLES E. CONGDON, M. D., Buffalo.

INASMUCH as one half of our gynecological operations are for the restoration of injuries, or the cure of the results of infection following parturition, it may be well to make an earnest and energetic inquiry as to the best methods to be used, that will be most effective in modifying this large percentage.

Most of us can remember when we depended solely, in our surgical work, upon antiseptics; we can also remember the results—infection, pus formation, pyemia and death from trivial operations. Today, in most of our best regulated clinics asepsis is the rule, with the result that there is no infection, no pus formation, scarcely a death, even in our major operations. If the same technique could be used in obstetrics that is, for instance, in a vaginal hysterectomy, I am convinced that no infection would take place.

If all lacerations of the cervix or of the vagina, both anterior along the urethra, as well as posterior in the sulci, or, as occasionally occurs, into the bowel, or tears of the vulva, were promptly repaired, a large measure of misery, both immediate and remote, would be spared our women and fewer would be brought to the gynecologist.

The question naturally arises, where lies the fault? and from my observations I am led to the belief that many of our good men allow lacerations to pass unannounced without repair, because of the fear that a knowledge of them would cause a reflection on their skill. I do not believe that it is a lack of knowledge on the part of physicians practising obstetrics. I have since learned from experience it is the ignorance, superstition and stupidity of the laity that is the sole cause of their morbidity in the majority of cases.

Very few households will permit the practice of well-organised and disciplined maternity work. This I learn not only from my own experience, but from that of many others. How are we to overcome this? It is very difficult and uphill work for a few men to teach the

---

1. Read before the Obstetrical Section, Buffalo Academy of Medicine, September 25, 1900.

masses; but it seems to me, that if we were to institute a united effort, that it would be possible to teach our patients in such a manner that they would forget their present ideas and consider us more than mere midwives, if we could teach them that they are to be cared for from the time of conception until involution; that we can do much to relieve the discomforts of pregnancy; that by frequent urinary analysis we may be able to render them less liable to eclampsia; that cleanliness is next to Godliness; that their beds should not be made or covered with old quilts or carpets, or with soiled linen, but with linen fresh from the iron; that the patient should have an enema sufficient to unload the bowel before the second stage of labor; that the vulva should be thoroughly shaved and scrubbed and kept closed with a sterile napkin; that in the primipara, with rare exception, lacerations occur; that irrigation, medicated or unmedicated, is harmful; that self examination is interdicted; that the physician is the better judge of the competency of the nurse; that the proper time for the resumption of household cares and duties does not depend on the number of days, but upon the condition of the patient, which should not be before involution has taken place; and, last, but not least, the patient should submit herself within two months after labor to a most careful examination, and if any defect is found it should be immediately repaired; if, I say, we would do all this, I am confident we would evolute at least a notch in the right direction.

1034 Jefferson Street.

